# Supplementary material for: Clomifene and Assisted Reproductive Technology in Humans Are Associated with Sex-Specific Offspring Epigenetic Alterations in Imprinted Control Regions
Source: Int J Mol Sci. 2022 Sep 9;23(18):10450. doi: 10.3390/ijms231810450 (PMC9499479; doi:10.3390/ijms231810450)
Supplement: Supplementary file 1 [file ijms-23-10450-s001.zip › ijms-1896319-supplementary/Figure S2.ART_ET_ICSI_outliersremoved.pdf]

ART ET/ICSI and Offspring Methylation

Change in Mean Methylation %

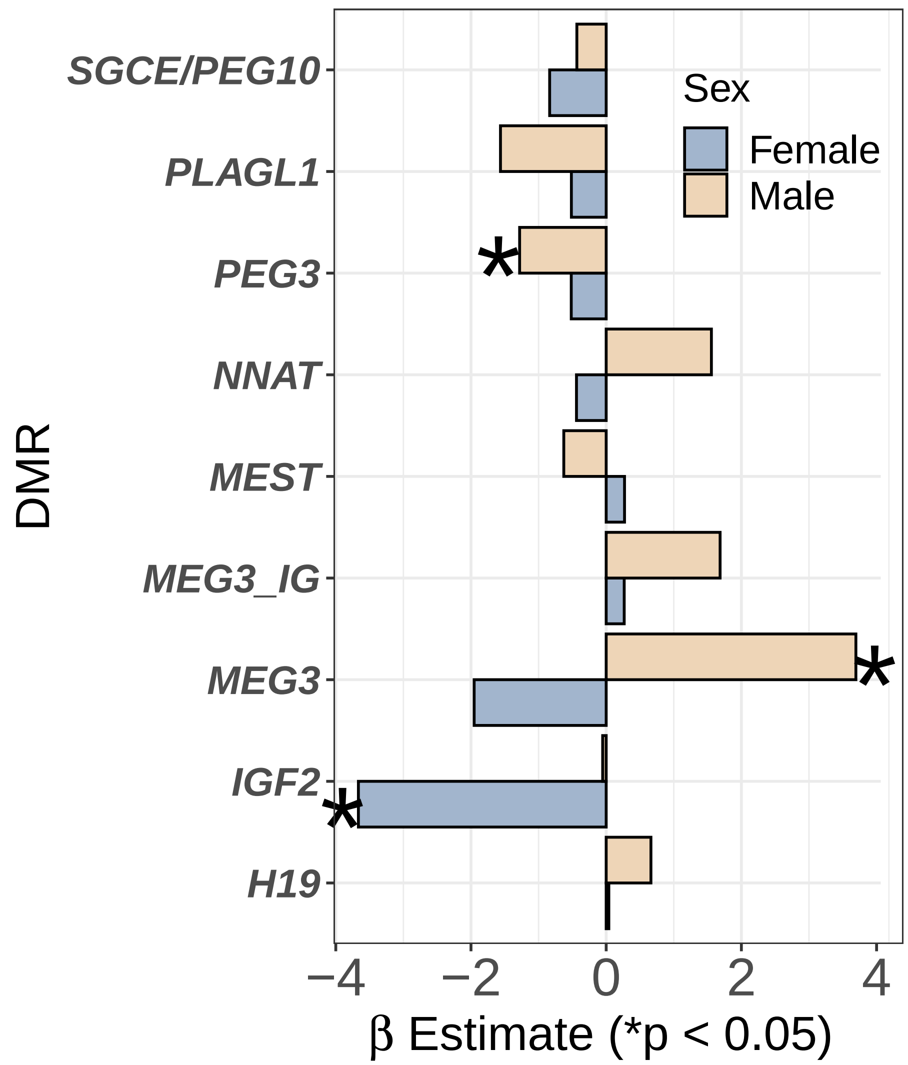

	Cases(n)	Controls(n)	Estimate(95%CI)	Pvalue
SGCE/PEG10−M	10	182	−0.43 (−1.99, 1.12)	0.582
SGCE/PEG10−F	12	**169**	−0.84 (−2.36, 0.69)	0.281
PLAGL1−M	9	**191**	−1.56 (−5.17, 2.05)	0.394
PLAGL1−F	13	**178**	−0.51 (−3.92, 2.89)	0.766
PEG3−M	8	**184**	−1.28 (−2.45, −0.11)	0.032
PEG3−F	**13**	**159**	−0.52 (−1.81, 0.78)	0.432
NNAT−M	9	**170**	1.56 (−2.51, 5.62)	0.451
NNAT−F	11	**157**	−0.44 (−3.82, 2.94)	0.797
MEST−M	10	**174**	−0.63 (−3.29, 2.03)	0.642
MEST−F	**12**	166	0.27 (−2.07, 2.61)	0.819
MEG3_IG−M	9	**229**	1.69 (−0.35, 3.72)	0.105
MEG3_IG−F	14	**194**	0.27 (−1.71, 2.24)	0.790
MEG3−M	11	**248**	3.69 (0.10, 7.29)	0.044
MEG3−F	**12**	227	−1.95 (−5.55, 1.65)	0.286
IGF2−M	6	146	−0.05 (−4.27, 4.17)	0.981
IGF2−F	9	143	−3.67 (−6.79, −0.55)	0.022
H19−M	6	**149**	0.66 (−2.06, 3.38)	0.631
H19−F	13	**150**	0.04 (−1.75, 1.83)	0.966

Linear regression for ART use for the index pregnancy and mean CpG methylation. We stratified models by sex and adjusted for pyrosequencing batch, pre-pregnancy maternal BMI, race, maternal age, diabetes, and parity. Estimates represent the mean percentage of methylation change associated with maternal use of ART (*p < 0.05). **Bolded** values in table represent outlier removal (+/- 3sd from mean).
