# Supplementary material for: Clomifene and Assisted Reproductive Technology in Humans Are Associated with Sex-Specific Offspring Epigenetic Alterations in Imprinted Control Regions
Source: Int J Mol Sci. 2022 Sep 9;23(18):10450. doi: 10.3390/ijms231810450 (PMC9499479; doi:10.3390/ijms231810450)
Supplement: Supplementary file 1 [file ijms-23-10450-s001.zip › ijms-1896319-supplementary/Figure S3.Clomifene_all.pdf]

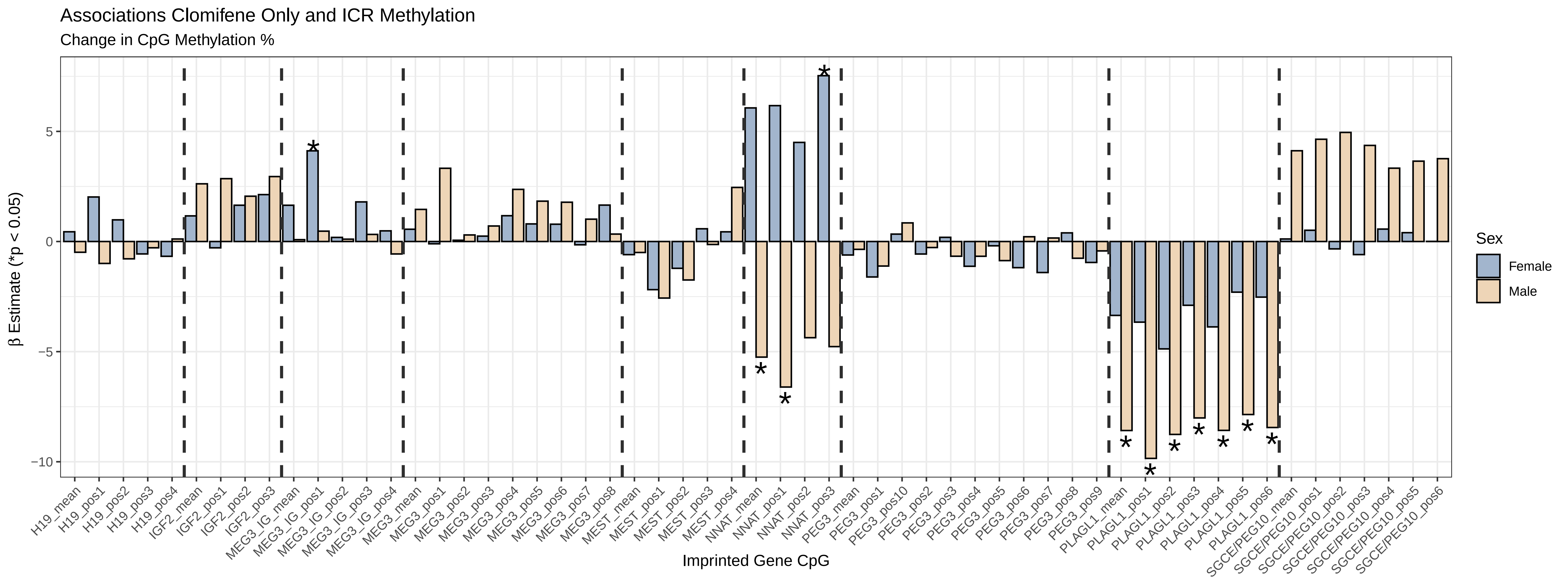

Linear regression for Clomifene only use for the index pregnancy and CpG methylation. We stratified models by sex and adjusted for pyrosequencing batch, pre-pregnancy maternal BMI, race, maternal age, diabetes, and parity. Estimates represent the mean percentage of methylation change associated with maternal use of Clomifene (*$p < 0.05$).
