# Supplementary material for: Clomifene and Assisted Reproductive Technology in Humans Are Associated with Sex-Specific Offspring Epigenetic Alterations in Imprinted Control Regions
Source: Int J Mol Sci. 2022 Sep 9;23(18):10450. doi: 10.3390/ijms231810450 (PMC9499479; doi:10.3390/ijms231810450)
Supplement: Supplementary file 1 [file ijms-23-10450-s001.zip › ijms-1896319-supplementary/Figure S4.Clomifene_outliersremoved.pdf]

Clomifene and Offspring Methylation

Change in Mean Methylation %

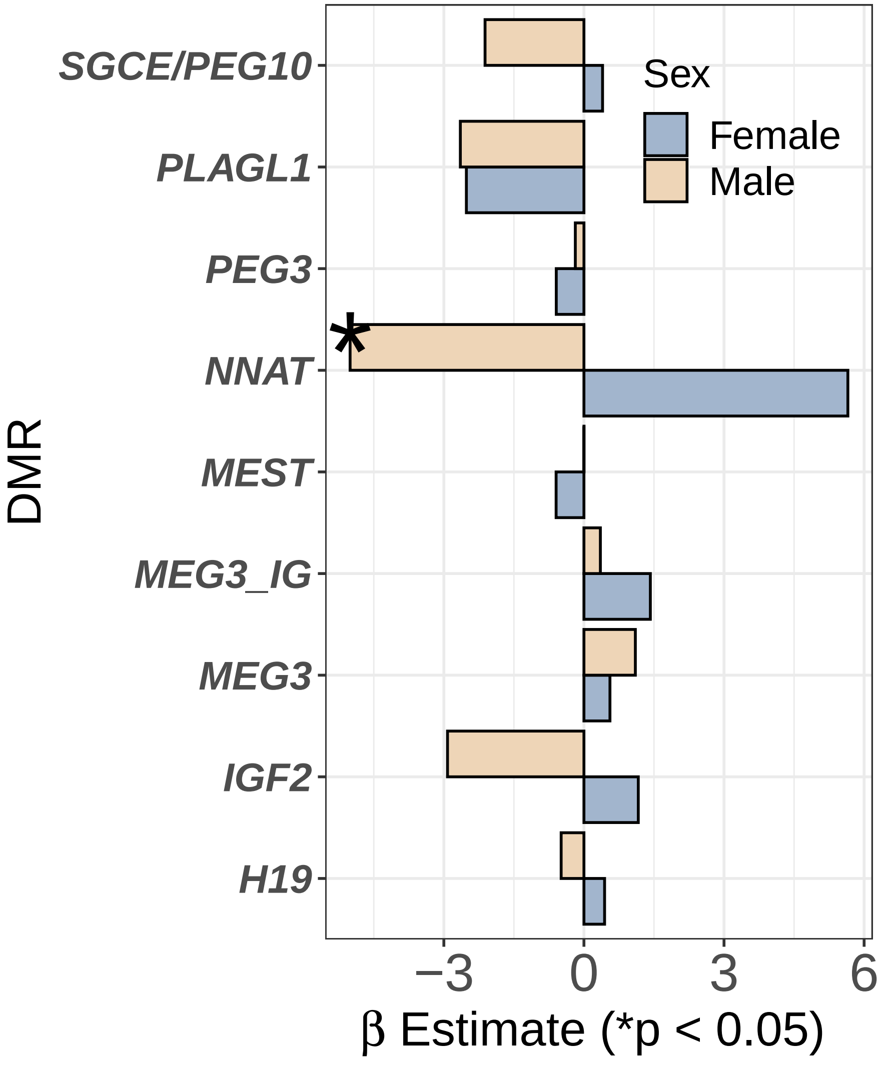

	Cases(n)	Controls(n)	Estimate(95%CI)	Pvalue
SGCE/PEG10−M	**11**	94	−2.12 (−4.75, 0.52)	0.114
SGCE/PEG10−F	7	**77**	0.40 (−2.56, 3.36)	0.788
PLAGL1−M	**10**	101	−2.65 (−8.10, 2.81)	0.337
PLAGL1−F	8	**86**	−2.52 (−8.66, 3.62)	0.416
PEG3−M	10	**95**	−0.18 (−1.57, 1.20)	0.791
PEG3−F	8	**70**	−0.59 (−2.28, 1.10)	0.485
NNAT−M	11	**83**	−5.01 (−9.80, −0.22)	0.041
NNAT−F	8	**66**	5.65 (−0.61, 11.91)	0.076
MEST−M	**10**	**90**	0.00 (−3.92, 3.92)	0.999
MEST−F	7	77	−0.60 (−5.23, 4.04)	0.798
MEG3_IG−M	11	**137**	0.35 (−3.35, 4.05)	0.851
MEG3_IG−F	10	**107**	1.42 (−1.39, 4.24)	0.318
MEG3−M	9	**157**	1.10 (−5.33, 7.53)	0.736
MEG3−F	10	134	0.56 (−4.05, 5.16)	0.811
IGF2−M	**9**	102	−2.92 (−7.12, 1.27)	0.170
IGF2−F	7	86	1.16 (−3.14, 5.46)	0.591
H19−M	9	92	−0.49 (−3.12, 2.14)	0.713
H19−F	6	83	0.44 (−2.22, 3.10)	0.741

Linear regression for Clomifene use for the index pregnancy and mean CpG methylation. We stratified models by sex and adjusted for pyrosequencing batch, pre-pregnancy maternal BMI, race, maternal age, diabetes, and parity. Estimates represent the mean percentage of methylation change associated with maternal use of Clomifene (*p < 0.05). Bolded values in table represent outlier removal (+/- 3sd from mean).
